# Protein-Polymer Matrices with Embedded Carbon Nanotubes for Tissue Engineering: Regularities of Formation and Features of Interaction with Cell Membranes

**DOI:** 10.3390/ma12193083

**Published:** 2019-09-21

**Authors:** Michael M. Slepchenkov, Alexander Yu. Gerasimenko, Dmitry V. Telyshev, Olga E. Glukhova

**Affiliations:** 1 Department of Physics, Saratov State University, Astrakhanskaya street 83, Saratov 410012, Russia; slepchenkovm@mail.ru; 2 Laboratory of Biomedical Nanotechnology, I.M. Sechenov First Moscow State Medical University, Bolshaya Pirogovskaya street 2-4, Moscow 119991, Russia; gerasimenko@bms.zone (A.Y.G.); d_telyshev@mail.ru (D.V.T.); 3 Institute of Biomedical Systems, National Research University of Electronic Technology MIET, Shokin Square 1, Zelenograd, Moscow 124498, Russia

**Keywords:** protein–polymer matrices, nanowelding, single-walled carbon nanotubes, point defects, absorption, laser radiation, cell membrane

## Abstract

This paper reveals the mechanism of nanowelding a branched network of single-walled carbon nanotubes (SWCNTs) used as a framework for the formation of protein–polymer matrices with albumin, collagen, and chitosan. It is shown that the introduction of certain point defects into the structure of SWCNTs (single vacancy, double vacancy, Stone–Wales defect, and a mixed defect) allows us to obtain strong heating in defective regions as compared to ideal SWCNTs. The wavelengths at which absorption reaches 50% are determined. Non-uniform absorption of laser radiation along with inefficient heat removal in defective regions determines the formation of hot spots, in which nanowelding of SWCNTs is observed even at 0.36 nm between contacting surfaces. The regularities of formation of layered protein–polymer matrices and the features of their interaction with cell membrane are revealed. All studies are carried out in silico using high-precision quantum approaches.

## 1. Introduction

At present, a 3D wireframe nanomaterial in the form of a branched network of multi-walled carbon nanotubes (MWCNTs) and SWCNTs is extremely popular in various fields, including biomedicine [1,2,3,4,5,6]. In recent years, 3D carbon nanotube (CNT) scaffolds have been especially demanded in the field of tissue engineering as a material for creating conductive scaffolds used for bone tissue regeneration [7]. The ability of 3D CNT scaffolds to stimulate the proliferation, maturation, and long-term survival of cardiomyocytes has already been proven [8]. An elastomeric scaffold developed based on the 3D CNT framework and polydimethylsiloxane stimulates the growth and electrophysiological maturation of cardiomyocytes, as well as the formation of functional syncytium [9]. 3D porous conductive CNT/polypyrrole scaffolds showed remarkable ability to regenerate astrocytes, which suggests their high potential as a neural prosthesis [10]. 

To create branched junctions of CNTs, one can use various methods and approaches, including direct growth [11,12,13], high-energy electron beam irradiation [14,15,16], forming junctions in strong electrical, optical, and thermal fields [17,18,19], as well as using atomic force microscopy [20]. The laser nanowelding technique seems to be the most promising method for obtaining high-quality 3D scaffolds from CNT junctions [21]. It was found that the key factors in nanowelding of MWCNTs are the degree of graphitization of MWCNTs, the irradiation time, the type of substrate, and others [21,22,23,24]. However, the degree of absorption of laser radiation by CNTs during welding at various wavelengths was not previously estimated. Also, the influence of the CNT structure, in particular CNT chirality, the presence of defects and their type on the nature of nanowelding and its quality was not taken into account. Knowledge of these regularities will allow a deeper understanding of the mechanism of splicing CNTs under the action of laser radiation in order to develop a controlled experimental method for obtaining 3D CNT scaffolds characterized by high conductive and strength properties. 

Taking into account such a variety of factors influencing the welding of CNTs within a single experimental study is a difficult task. In addition, a detailed study of the CNT welding mechanism requires studies of the physical processes occurring in the structure of CNTs at the atomic and quantum levels. Such studies can only be carried out using computer simulation methods. The first works in this direction appeared at the beginning of the 21st century [25,26]. In these first works, the nanowelding of SWCNTs of various shapes under the influence of ion and electron irradiation was simulated using molecular dynamics. In the last decade, the process of nanowelding is studied using simulation methods from different angles. On the one hand, nanowelding is modeled as a result of simple Joule heating [18,27], and on the other hand, as a result of the formation of X, Y, and T-shaped SWCNT junctions due to the melting of silver nanoparticles [28,29,30]. In the second case, the nanoparticles play the role of a “bonding adhesive” at the junction of the SWCNTs. It has already been repeatedly shown that a welding of SWCNT framework occurs during strong heating to 1300–1700 K, which ensures a junction of the SWCNT open ends with each other and with individual SWCNTs [2,18]. When heated to 2500–3500 K, the SWCNTs do not just connect to each other, but weld seamlessly, forming a new X-shaped structure [17,31]. The main question in this case is the SWCNT nanowelding mechanism. As already mentioned, a strong heating of the SWCNTs is necessary to start the nanowelding mechanism. Today, several methods of heating are known. One of the promising methods is a welding using laser pulsed irradiation. Under the action of a laser beam, silver nanoparticles melt and connect the CNTs together. Another example is the method of laser nanowelding of bundles of double-walled CNTs, when fragments of CNTs resulting from the partial destruction of the outer shells of multilayer CNTs act as solder [32]. However, to date, there is no information about the dependence of the degree of absorption of electromagnetic waves by nanotubes on their chirality and the presence of defects, and also about the influence of these factors on the nanowelding process. 

Another relevant direction of a study of the properties and applications of 3D nanomaterial in the form of a branched network of MWCNTs and SWCNTs is the use of these materials for the subsequent creation of protein and polymer matrices on their basis. Such matrices are already actively synthesized and in demand in various fields of biomedicine [33,34], including in the field of tissue engineering [34]. It has already been proven that these matrices are biocompatible, but it is not yet known how exactly such matrices contact cell membranes and what effect they have on them. One of the most promising candidates for creating protein and polymer matrices are albumin, collagen and chitosan. Albumin is already being used for neural tissue engineering applications [35], for lungs tissue engineering applications [36], for engineering functional cardiac tissues [37], and also to create a potential degradable tissue scaffold [38]. Collagen-based biomaterials function as cell scaffolds and replace the native extracellular matrix [39]. A collagen scaffold can be applied in tissue engineering, including nerve, bone, cartilage, tendon, ligament, blood vessel and skin [40,41]. The natural abundance, cost-effectiveness, biodegradability, biocompatibility and the ability to heal wounds have made chitosan a very popular polymer for tissue engineering and implantation [42,43]. 

In this work, we perform in silico study of the formation of a branched network of SWCNTs as a result of nanoscale welding of SWCNTs under the action of laser irradiation. The most effective frequency of laser irradiation for welding of SWCNTs is revealed. Modeling of the formation of layered protein–polymer matrices with a branched network of SWCNTs as a framework and albumin, collagen and chitosan as fillers is carried out. Some regularities of interaction of polymer–protein matrices with the cell membrane are also studied.

## 2. Computational Details

To understand the nature of CNT nanowelding, we considered the features of the interaction of the defective regions of SWCNTs and the open ends of SWCNTs with laser radiation in the ultraviolet (UV)–visible–infrared (IR) range. As was mentioned in the introduction, welding occurs during strong heating [2,18] precisely due to open ends, as well as due to the partial destruction of defective CNT regions [32]. It should also be noted that defective regions do not efficiently remove heat, so during general heating of the nanostructure, these regions overheat more noticeably and may eventually collapse. 

Let us consider the absorption of energy in the case of normal incidence of a plane electromagnetic wave. To do this, we calculate the absorption coefficient using Maxwell’s theory and quantum-mechanical approaches. The SWCNT array acts as the interface between two media, each of which is a vacuum. That is, we are considering the process of interaction of a SWCNT network with an incident wave, which passes from a vacuum through a SWCNT network again into a vacuum. We consider waves with the vector **E** directed along the CNT axis and perpendicular to it. The absorption coefficient is determined by the equation:

(1) $$A=1-{\left|R\right|}^{2}-{\left|T\right|}^{2},$$

where ${\left|R\right|}^{2}$ and ${\left|T\right|}^{2}$ are the squares of the moduli of the complex reflection and transmission coefficients. For the case of normal wave incidence, the expressions for *R* and *T* coefficients are written as follows [44]:

(2) $$R=\frac{-\sigma_{\alpha \beta }Z_{0}}{2+\sigma_{\alpha \beta }Z_{0}},T=\frac{2}{2+\sigma_{\alpha \beta }Z_{0}},$$

where $Z_{0}$ is the wave resistance in free space $Z_{0}=120\pi$ Ohm; $\sigma_{\alpha \beta }$ is an element of the complex optical conductivity tensor (α and β indices coincide in the direction of the axis of the nanotube or perpendicular to it). The elements of the complex optical conductivity tensor $\sigma_{\alpha \beta }(\Omega )$ were calculated using the Kubo–Greenwood formula that defines the conductivity as a function of photon energy Ω [45,46]:

(3) $$\sigma_{\alpha \beta }=\frac{2e^{2}\hbar }{im_{e}^{2}S_{cell}}\frac{1}{N_{k}}\sum_{k\in BZ}^{N_{k}}\sum_{m,n}\frac{{\hat{P}}_{\alpha }^{nm}(k)\cdot {\hat{P}}_{\beta }^{nm}(k)}{E_{n}(k)-E_{m}(k)+\Omega +i\eta }\times \frac{f_{\beta }[E_{n}(k)-\mu ]-f_{\beta }[E_{m}(k)-\mu ]}{E_{n}(k)-E_{m}(k)},$$

where $f_{\beta }(E)=1/(1+\exp[\beta (E-\mu )])$ is the Fermi–Dirac function with chemical potential *μ* and the inverse of thermal energy $\beta =1/k_{B}T$; $S_{cell}$ is the area of the super-cell; $N_{k}$ is the number of k-points needed to sample the Brillouin zone (BZ); ${\hat{P}}_{\alpha }^{nm}(k)$ are the matrix elements corresponding to the *α*-component of the momentum operator vector; ${\hat{P}}_{\beta }^{nm}(k)$ are the matrix elements corresponding to the *β*-component of the momentum operator vector; $m_{e}$ and e are the free-electron mass and electron charge; $E_{n}(k)$ is the sub-band energy in the valence band, $E_{m}(k)$ is the sub-band energy in the conduction band. The spin degeneracy is already taken into account in the above equations by factor 2, η is a phenomenological parameter which characterizes the processes of electron scattering. To calculate the momentum matrix elements ${\hat{P}}_{\alpha }^{nm}(k)$ we used the well-known expression substitution $\hat{P}(k)\to (m_{e}/\hbar )\nabla_{k}\hat{H}(k)$, where $\hat{H}(k)$ is the Hamiltonian. The Hamiltonian was constructed as part of the self-consistent charge density functional tight-binding (SCC DFTB) method. All calculations were performed using open source Kvazar [47] and DFTB + package [48]. The use of the SCC DFTB method instead of ab initio methods is due to the polyatomicity of the supercells of the studied SWCNT. For example, supercells of chiral SWCNTs contain 500–1324 atoms. We also note that Equation (2) is valid for thin films whose thickness is much less than the wavelength. These equations were obtained previously for composite thin films [44] and well tested.

To simulate the nanotube nanowelding process, the nonequilibrium molecular dynamics method with the adaptive intermolecular reactive bond order (AIREBO) force field was used [49]. This method is implemented in open source Kvazar. The simulation time step was 0.1 fs. Polymer–protein matrices based on a SWCNT network were simulated by means of the coarse-grained approach using the MARTINI force field [50] and the GROMACS program [51]. 

## 3. Results and Discussion

In this work, we consider chiral and non-chiral SWCNTs with a diameter of 0.6–2 nm, which are most often synthesized: (i) non-chiral zigzag SWCNTs (m, 0) with m = 13, 14, 16, 20, 23, 32 and armchair SWCNTs (m, m) with m = 4, 12, 15, 20; (ii) chiral SWCNTs (11,10), (14,4), (12,6), (12,8), (23,6) and (9,4). That is, semiconductor SWCNTs account for ~69% of the total number of SWCNTs under consideration. This fact is completely consistent with the experimental data, according to which semiconductor SWCNTs are always composed of ~2/3 from an array of synthesized SWCNTs. Previously, the authors of this work showed that the nanowelding of SWCNTs occurs in those regions of SWCNTs where there is a defect or several defects. In particular, the formation of a tree-like structure from SWCNTs due to the formation of T-shaped contacts was shown using the molecular dynamics method [2]. Similar T-shaped SWCNT structures are formed due to the formation of covalent bonds between the open ends of one SWCNT and the atoms of the defective region of another SWCNT. We assume that the reason for the formation of covalent bonds between SWCNTs is the nonuniform absorption of laser radiation energy, which inevitably leads to nonuniform heating of SWCNTs and the appearance of “hot spots” in which nanowelding occurs. The basis of our assumptions is a series of works in which the strong influence of various defects on the temperature distribution in nanotubes is proved. For example, J. Park et al. have shown that the temperature can increase by several hundred degrees in the defect region, and the thermal conductivity of the defective regions drops sharply [52]. It was found that such defects as Stone–Wales and vacancies strongly affect the thermal conductivity of a CNT network. They prevent the free propagation of phonons reducing thermal conductivity in these local regions by 2–10 times [53,54]. Another paper by M. Chang et al. [55] demonstrated the presence of a large temperature gradient due to a drop in thermal conductivity in defective regions, which inevitably leads to the appearance of heat localization. It was also noted there that the presence of local regions of high heating is characteristic of all low-dimensional structures, which was previously observed for silicon and aluminum wires of submicron diameter [56,57]. The reason for this phenomenon is (i) the scattering of phonons, the mean free path of which decreases significantly due to topological defects, and (ii) the phonons are limited in the direction of propagation in low-dimensional structures. This leads to the localization of heat. In this work, the nonuniform absorption of laser energy is caused by the presence of defects in the atomic structure. To verify our assumption, we first investigated the regularities of absorption of electromagnetic wave energy by the defective SWCNTs in the UV–visible–IR range using Equations (1)–(3) and the SCC DFTB method. 

### 3.1. Absorption of Electromagnetic Wave Energy by Defect-Free and Defective SWCNTs

**Figure 1:**
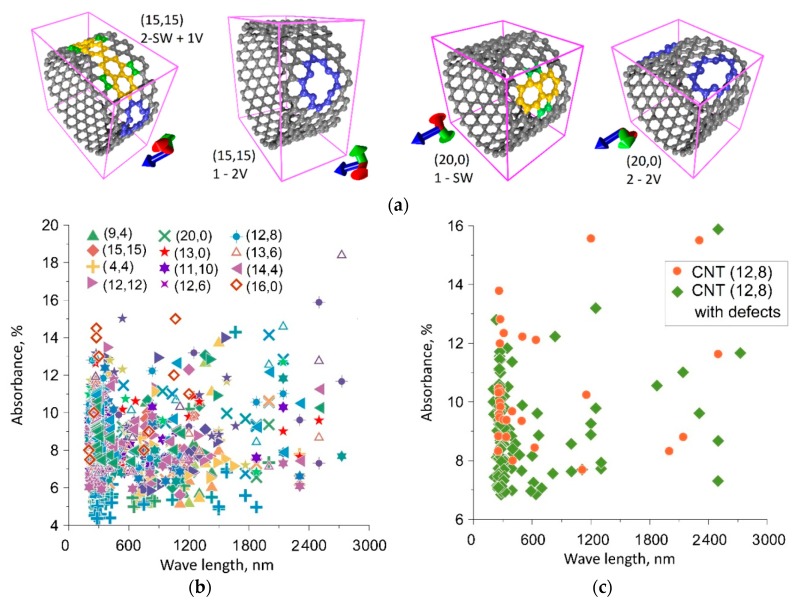
Absorption of the energy of electromagnetic waves by defective single-walled carbon nanotubes (SWCNTs): (**a**) Examples of atomistic models of defective SWCNTs (lilac box corresponds to a supercell); (**b**) distribution of the absorption coefficient maxima for the defective SWCNTs with a diameter of 0.6–2 nm; (**с**) distribution of the absorption coefficient maxima for defect-free and defective SWCNT (12,8).

We have constructed atomistic models of SWCNTs with point defects, such as single (1V) and double (2V) vacancies, Stone–Wales (SW) defects and an SW + 1V mixed defect. As an example, Figure 1a shows supercells of two SWCNTs with various point defects: SWCNT (15,15) with a mixed defect consisting of two SW defects and one 1V defect, SWCNT (20,0) with two 2V defects, SWCNT (20,0) with a single SW defect, SWCNT (15,15) with a single 2V defect. Heptagons are marked in yellow, atoms of adjacent pentagons are marked in green, and atoms in a region with 1V and 2V vacancies are marked in blue. The supercells of the defective SWCNTs under study were optimized and energetically favorable atomistic models of SWCNTs corresponding to the equilibrium structure were revealed. To calculate the optical conductivity and absorption coefficient, we constructed a model of a thin film of parallel SWCNTs located at a distance of ~0.5 nm. At this distance, the electron clouds of adjacent SWCNTs do not affect each other and, thus, we calculate the energy absorption of an individual SWCNT. Since the thickness of such a film of SWCNTs is no more than 2 nm, the range of the studied waves was 10–3000 nm. The wave vector is normal to the film. The calculated values of the absorption coefficient for SWCNTs of various diameters and chiralities are presented in Figure 1b. Since a lot of calculated data were obtained, this figure shows the results for only 12 SWCNTs, which are most often synthesized and have diameters in a wide range of 0.6–2 nm. Based on the calculation results, including data presented in Figure 1b, we can say that there are three wavelength regions in which the main number of absorption maxima is concentrated for all types of SWCNTs. These are wavelength ranges of 200–400 nm, 650–800 nm and 900–1150 nm. In the far infrared wavelength range of 1800–2400 nm, a certain number of absorption maxima is also observed, but only for certain types of defects and not for all types of SWCNTs. In order to evaluate exactly how defects in the atomic structure of SWCNTs affect the frequency distribution of absorption maxima, we calculated the absorption spectra of defect-free SWCNTs. Analyzing all the types of SWCNTs under study, one can conclude that the appearance of defects leads to the appearance of new absorption peaks, and at some wavelengths it also leads to an increase in the absorption coefficient. One such example is given for a SWCNT (12,8) in Figure 1c. Based on the calculated data, we can say that defective regions of SWCNTs are capable of absorbing more energy from external radiation than defect-free regions. And besides, defective regions are capable of absorbing energy in a wider range of wavelengths than defect-free ones. 

**Figure 2:**
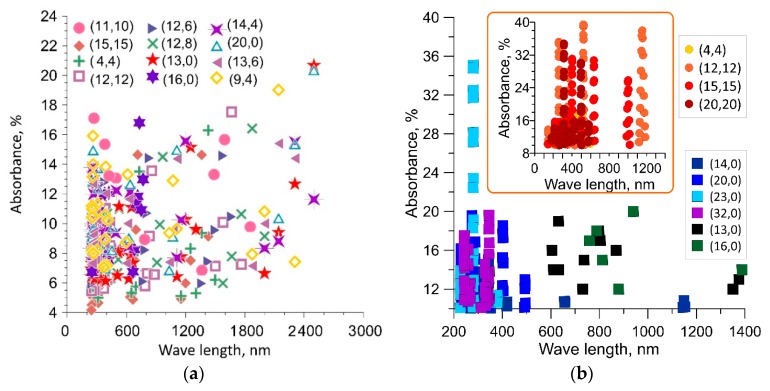
Distribution of absorption maxima for SWCNTs of different diameters: (**a**) SWCNTs of different chiralities; (**b**) achiral SWCNTs (circles correspond to armchair SWCNTs, squares—zigzag SWCNTs).

The next step in studying the absorption capacity of SWCNTs was to study the regularities of absorption of electromagnetic wave energy by the open ends of SWCNTs of finite length. The above calculation data were obtained for SWCNTs of infinite length, since we applied periodic boundary conditions. In this connection, a logical question arises: How do short SWCNTs absorb energy and what is the role of the open ends of SWCNTs in this case? To answer this question, we investigated SWCNTs with a length of several supercells. The maximum length of the considered short SWCNTs was 38–40 nm. The choice of this length is due to the large number of atoms in chiral SWCNTs, namely, 6000–8000. Nanotubes with a large number of atoms cannot be investigated by quantum methods. To calculate the absorption coefficient of electromagnetic waves by the open ends of SWCNTs, we built a model of a film ~40 nm thick corresponding to the length of SWCNTs. SWCNTs were arranged in parallel, wherein the outer surface of SWCNTs was formed by the open ends. The wave vector of the incident wave was directed normal to the film, that is, along the axes of the SWCNTs. This model allows us to calculate the absorption properties of the open ends of the SWCNTs. However, since the film thickness was 40 nm, the considered wavelength range was 100–3000 nm. Figure 2 shows the distribution diagrams of the absorption maxima: Figure 2a—for SWCNTs of various chiralities and diameters, Figure 2b—for achiral semiconductor SWCNTs and metal SWCNTs (in the inset). In contrast to extended SWCNTs that absorb energy by the side surface, the open ends of short SWCNTs provide significantly greater energy absorption. Figure 2a shows that in the UV region, the absorption maxima are concentrated in the wavelength range of 200–400 nm; in the visible region, the absorption maxima are concentrated in the wavelength range of 600–700 nm. In the IR region, there is no such pronounced interval; the absorption maxima are distributed over the entire wavelength range of 800–3000 nm. The largest absorption maxima are observed at wavelengths of 1700, 2200 and 2300 nm. The inset in Figure 2b shows the distribution diagrams of absorption maxima above 10%. In the case of metal SWCNTs, the absorption reaches even 40% at wavelengths of 650–750 nm and 1050–1250 nm. In the case of semiconductor SWCNTs, the absorption reaches 36% only in the UV range at wavelengths of 250–350 nm. 

### 3.2. The formation of A Network of SWCNTs under The Action of Laser Radiation

**Figure 3:**
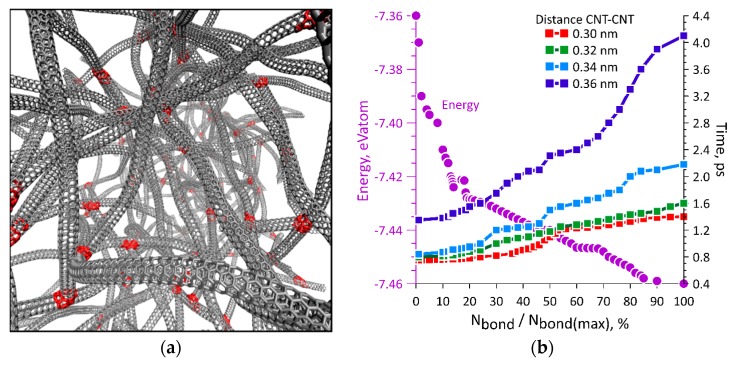
Formation of a carbon network: (**a**) A branched network of SWCNTs (4,4) with red regions corresponding to the nanowelding points; (**b**) plots of change in the energy of the network of SWCNTs (purple curve) and time of the formation of covalent bonds.

Further, by the example of the SWCNTs (4,4), we studied the process of carbon network formation as a result of absorption of laser beam energy. A carbon network is a branched structure of SWCNTs. The choice of SWCNTs (4,4) is due to their small diameter, which makes it possible to introduce a larger number of SWCNTs into the model of SWCNT network with the same total number of atoms. Figure 3a shows a fragment of a network of SWCNTs (4,4). In total, the network model included 258 SWCNTs with a length of 5–20 nm. SWCNTs were located in a periodic box with size of 65 nm × 45 nm × 40 nm. The total number of atoms in the model was 174,776. SWCNTs were arranged randomly taking into account the van der Waals interaction between them, but without the formation of covalent bonds between the SWCNTs. The density of the created SWCNT network was 60 kg/m^3^. After that, defects of various types were created in those places where the SWCNTs were located at a distance of 0.2–0.4 nm. The introduction of defects is necessary for the implementation of the nanowelding process. The nanowelding process was implemented by non-uniform temperature distribution. The interaction of an SWCNT array with a laser beam consists of non-uniform absorption of electromagnetic wave energy, that is, in non-uniform heating. According to the absorption coefficients calculated by us, in the defective regions and in the vicinity of the open ends of the SWCNTs, the temperature was 16%–20% higher than the temperature in defect-free regions. Further, a molecular dynamics simulation of the welding process was carried out. The red color in Figure 3a denotes the nanowelding points at the places of jointing open ends of the SWCNTs with defective SWCNTs, as well as at the junctions of the SWCNTs with each other due to the interaction between atoms of the defective regions. Figure 3b shows a plot of change in the carbon network energy (in units of energy per atom eV/atom, the violet axis and the violet curve) during the formation of covalent bonds between SWCNTs. The number of formed bonds is given as a percentage of the total possible number of covalent bonds for a given network. It can be seen that the energy decreases nonlinearly as the formed bonds increase. The plots of time of the formation of covalent bonds between the SWCNTs (red, green, blue, and dark blue curves) versus distance between them are also presented in Figure 3b. As expected, nanowelding most rapidly occurs at a small distance between the SWCNTs. The presented regularity of energy change (purple curve) is characteristic for all cases, regardless of the distance between the tubes, and is the result of averaging a large number of numerical experiments. The energy of the structure and the time of formation of covalent CNT–CNT bonds are directly linked. This can be seen from Figure 3b. The more CNT–CNT bonds are formed, the lower the energy, which means that the process of forming covalent bonds between the CNTs in the nanowelding process is energetically beneficial. According to our calculations, in general, a few picoseconds are enough to form bonds. 

### 3.3. The Formation of Layered Protein–Polymer Matrices

**Figure 4:**
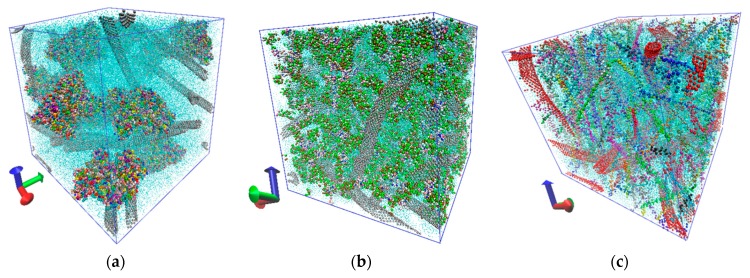
Coarse-grained models of individual layers of protein–polymer matrices: (**a**) SWCNT–albumin; (**b**) SWCNT–collagen; (**c**) SWCNT–chitosan.

Earlier, we studied the regularities of formation of protein–polymer matrices based on a network of SWCNTs, albumin, collagen, and chitosan [34]. In this paper, we study the regularities of formation of a layered protein–polymer matrix of three layers, including CNT-albumin, CNT-collagen, and CNT-chitosan layers. For this, as in the previous study [34], we used the coarse-grained modeling method. Coarse-grained models of individual matrix layers are shown in Figure 4. Figure 4a shows a periodic box of the SWCNT + albumin protein matrix, where SWCNTs with a diameter of 1.6 nm and albumin macromolecules are located. The size of the periodic box was 30 nm × 30 nm × 30 nm. The periodic box contains 19,827 grains of SWCNTs and 25,970 grains of five albumin molecules. Grains of water molecules are marked in blue. The box contains 200,000 grains of water. In total, the structure contains 245,797 grains. The periodic box of the SWCNT + collagen protein matrix is shown in Figure 4b, where 160,000 water grains, 19,827 SWCN grains and 56,498 collagen grains are present. The periodic box of the SWCNT + chitosan polymer matrix is shown in Figure 4c. Since chitosan chains are very numerous, SWCNTs are marked in bright red, and chitosan in green, pink, and blue. This box contains 200,000 water grains and 9,237 chitosan grains. All structures were optimized with a thermostat 310 K. 

**Figure 5:**
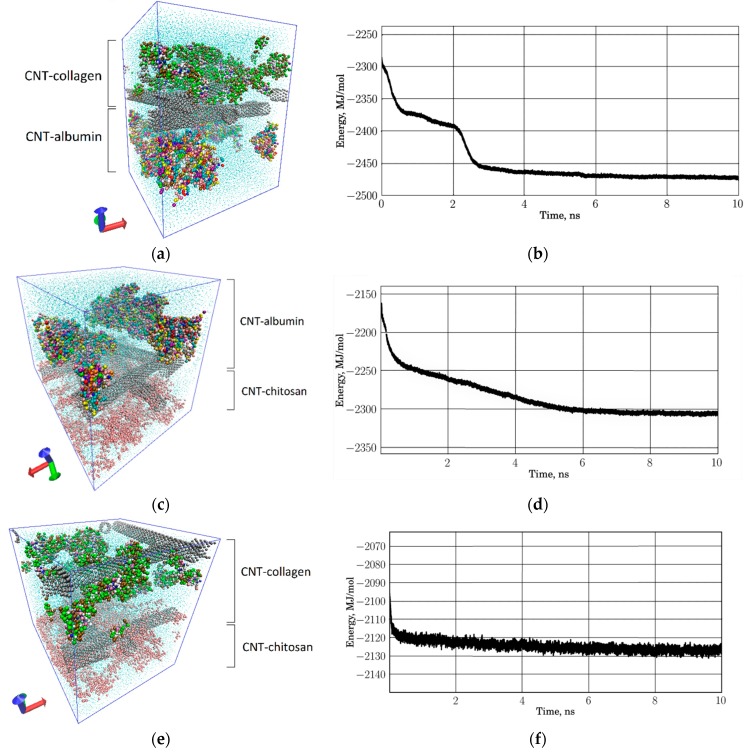
Layered 2D protein-polymer matrices: Coarse-grained model (**a**) and energy of albumin–collagen structure (**b**); coarse-grained model (**c**) and energy of albumin–chitosan structure (**d**); coarse-grained model (**e**) and energy of collagen–chitosan structure (**f**).

Next, a layered structure of SWCNTs and natural polymers was built. The construction principle was as follows. Two different layers were taken and combined into a new periodic box, which is translated only in two directions along X and Y. The distance between adjacent layers was taken equal to the van der Waals distance between the components forming the layers. Molecular dynamic optimization was carried out at a temperature of 310 K. The box was also optimized in two directions. The result was a periodic box of a two-layer structure. During optimization, the components at the boundary of two layers mutually penetrated into the adjacent layer. An example of a layered protein matrix of SWCNTs, albumin, and collagen is shown in Figure 5a. Coarse-grained models of SWCNTs are shown in gray. Layers with different proteins are marked. The energy of such a structure changed during the formation and its plot is shown in Figure 5b. As the components of the layers at the boundary were optimized, the energy fell until it stabilized and stopped changing, which indicates the stabilization of the two-layer protein matrix. Similarly, a model of a two-layer protein–polymer matrix was constructed from SWCNTs, albumin, and chitosan. It is presented in Figure 5c. To make it easier to see both layers, all the chitosan threads are depicted in pink (bottom layer). SWCNTs are marked in gray. The plot of energy change is presented in Figure 5d, which shows the decrease in energy during the optimization of the structure. In contrast to the previous case, when the two-layer protein matrix stabilized in 4 ns, in this case the stabilization time was 6 ns. Another two-layer protein–polymer matrix is shown in Figure 5e, where the SWCNT–collagen layer acts as a protein layer. In this case, the structure optimization was already completed within the first 2 ns, as can be seen from the plot in Figure 5f. This is explained by the fact that collagen contacts to SWCNTs rather tightly; therefore, large changes are not observed at the boundary of two layers. The thickness of each layer was ~11.5 nm in all cases; the size of the periodic box in the XY plane was ~19 nm × 19 nm in all cases. 

### 3.4. The Interaction of Layered Protein–Polymer Structures with the Cell Membrane

**Figure 6:**
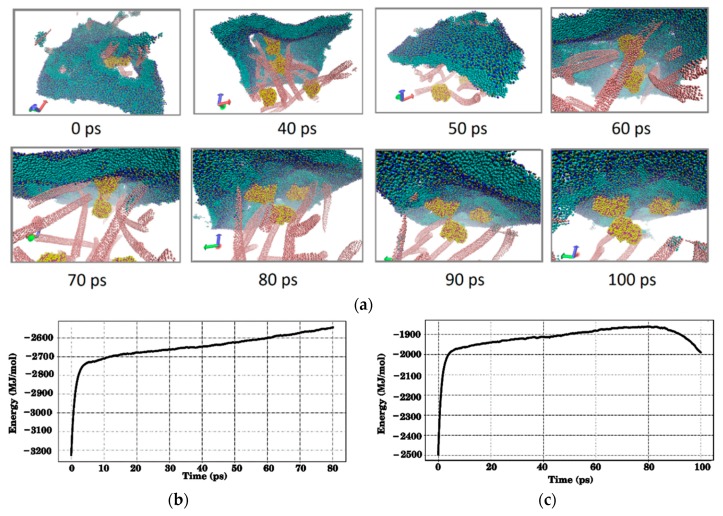
The interaction of the membrane with the SWCNT-albumin protein matrix: (**a**) Snapshots of the process of detachment of the membrane from the surface of the matrix; (**b**) a plot of change in the energy of the matrix + membrane system when the membrane falls on the matrix; (**c**) a plot of change in the energy of the matrix + membrane system when the membrane detaches from the matrix and moves away from it (SWCNTs are marked in pink, albumin molecules are marked in yellow and red, and the membrane is marked green and blue).

A layer of phospholipid molecules was taken as a cell membrane. The membrane is a layer of 8,450 coarse-grained molecules of DPPC (Dipalmitoylphosphatidylcholine). The total number of coarse grains in the membrane is 101,400. The length and width of the membrane are 50 nm, and the thickness is 4 nm. First, we studied the process of interaction of a CNT–albumin protein matrix with a membrane (see Figure 6). The model shown in Figure 4a was used. The process of contact of the membrane with the matrix was modeled as follows. The membrane moves to the matrix with an average blood flow velocity of 0.5 m/s, which simulates the collision of blood elements, for example, an erythrocyte with the surface of the CNT-albumin protein matrix. The surface of the matrix is sticking out SWCNTs with albumin molecules. SWCNTs are arranged at different angles in an arbitrary way. Additionally, part of the SWCNTs is strictly vertically oriented. When the membrane falls on the surface of the matrix, it is pierced by SWCNTs. Then the membrane rises, returning to its original position. This simulates the rolling of blood elements on the surface of a protein matrix. Figure 6a shows snapshots of the process of detachment of the membrane from the surface. The point in time 0 ps in the figure corresponds to the moment when the membrane begins to detach. The total time was 100 ps. In Figure 6a, the SWCNT network is presented in pink, the grains of albumin molecules have yellow and red colors, and the hydrophilic parts of the DPPC molecules are shown in green and hydrophobic heads in blue. For ease of viewing, water grains are removed. Molecular dynamic modeling of this process showed that three of the five albumin molecules are captured by the membrane and “stuck” to its surface. This is due to the fact that these molecules are directly on the surface. Two other albumin molecules located deep in the SWCNT framework remain in the matrix. Snapshots in Figure 6a also demonstrate that within 90–100 ps, the holes formed in the membrane are almost completely drawn out. Figure 6b,c show plots of the total energy of the matrix + membrane system during the falling membrane onto the surface of the matrix (see Figure 6b) and during the detachment of the membrane with its subsequent distancing (see Figure 6c). During contact and subsequent interaction, when some SWCNTs pierce the membrane, the energy increases sharply during the first ten picoseconds (see Figure 6b). With a further membrane falling, the energy increases, but more slowly. A similar behavior of energy is observed in the case of detachment and reverse movement of the membrane (see Figure 6c). In this case, a sharp increase in the energy is due to the beginning of the process of detachment of some albumin molecules from the carbon framework. Within 90 ps, the process of detachment of albumin molecules from SWCNTs finishes, therefore, an increase in energy replaced by its decrease. And further, the decrease in energy continues until the membrane structure is completely restored. 

**Figure 7:**
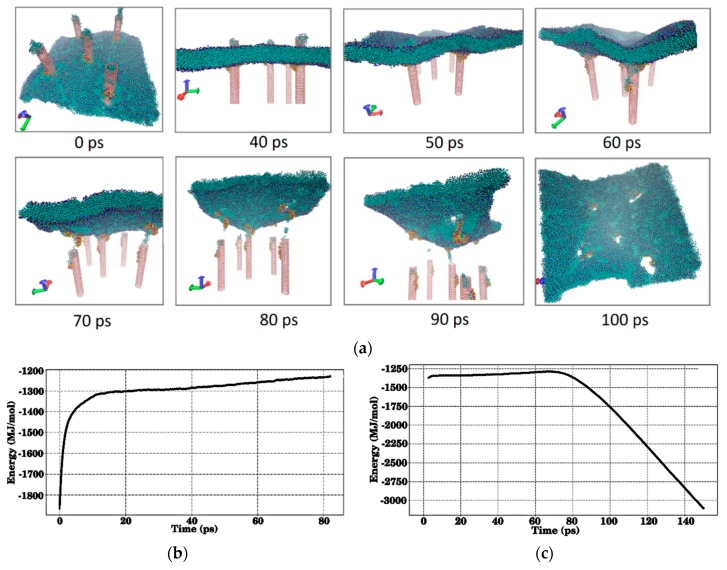
The interaction of the membrane with the SWCNT-collagen protein matrix: (**a**) Snapshots of the process of detachment of the membrane from the surface of the matrix; (**b**) a plot of change in the energy of the matrix + membrane system when the membrane falls on the matrix; (**c**) a plot of change in the energy of the matrix + membrane system when the membrane detaches from the matrix and moves away from it (SWCNTs are marked in pink, albumin molecules are marked in yellow and red, and the membrane is marked in green and blue).

Similarly, the falling of the membrane onto the SWCNT–collagen protein matrix was studied. Based on the experience of studying the contact of the membrane with the SWCNT-albumin matrix, we took a fragment of the model imaged in Figure 4b, where the SWCNTs are vertically oriented relative to the membrane. This was done due to the fact that the most important is the piercing of the membrane by SWCNTs and the process of the membrane subsequent recovery. Collagen molecules are directly located on the SWCNTs. The simulation result of the process of membrane falling onto the matrix is shown in Figure 7a. The initial moment is the moment when the membrane is pierced by SWCNTs in contact with the matrix, and then the process of detachment of the membrane begins. Within 70 ps, a complete detachment of the membrane occurs, wherein some of the collagen molecules remain on the membrane. The membrane is deformed and bears traces of contacts with SWCNTs in the form of holes. Over the next 30 ps, the holes gradually tighten and by the point in time 100 ps they are almost gone. It can be concluded that the membrane is not damaged, since only a few DPPC molecules remain on the SWCNTs after contact with the membrane. All this process of interaction of the membrane with the matrix can be traced by change in the energy of the entire membrane + matrix system. A plot of energy in Figure 7b for a case of membrane falling onto the matrix clearly shows that when the membrane penetrates the SWCNTs, the energy of the entire system increases sharply. This occurs in the first 10 ps. Further, the energy slowly increases, which corresponds to the further threading of the membrane on the SWCNTs. The energy of the matrix + membrane system from the beginning of the membrane rise is shown in Figure 7c. It is completely identical to the visual snapshots of Figure 7a. During the first 70 ps after the beginning of detachment, the energy practically does not change, increasing very slowly. And then it decreases sharply when the membrane detached from SWCNTs. Further molecular dynamics modeling (after 100 ps) shows that the membrane completely restores its initial structure and shape. 

**Figure 8:**
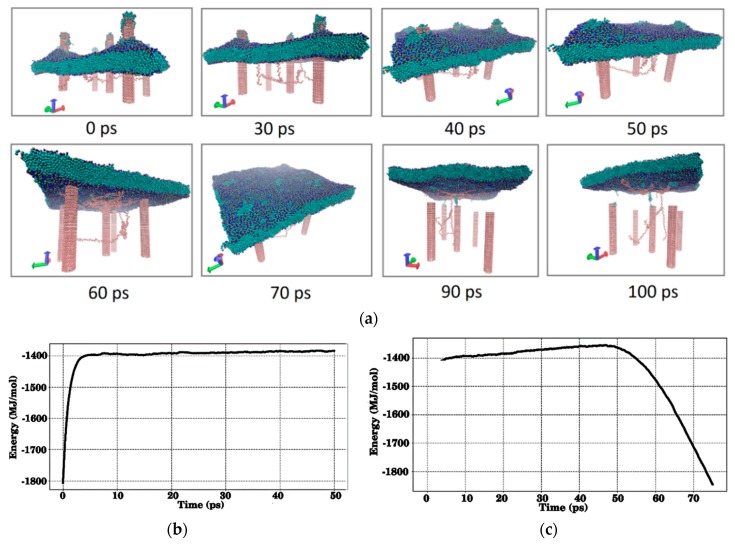
The interaction of the membrane with the CNT–chitosan polymer matrix: (**a**) Snapshots of the process of detachment of the membrane from the surface of the matrix; (**b**) a plot of change in the energy of the matrix + membrane system when the membrane falls on the matrix; (**c**) a plot of change in the energy of the matrix + membrane system when the membrane detaches from the matrix and its backward movement from the matrix (CNTs and chitosan filaments are marked in pink, the membrane is marked in green and blue).

And, finally, the process of membrane contact with a fragment of the SWCNT–chitosan matrix was studied. As in the previous case, a small fragment was taken with vertically oriented SWCNTs with respect to the membrane. The entire process of membrane contact with SWCNTs and chitosan threads between them is shown in Figure 8. The change in the SWCNT–chitosan structure is shown in Figure 8a, the change in energy during the membrane falling onto the matrix is shown in Figure 8b, and the change in energy during the detachment of the membrane is shown in Figure 8c. In general, the process is completely identical to the previous case. Some of the chitosan molecules “stick” to the membrane; some molecules remain in the matrix. Holes in the membrane from contact with nanotubes disappear within 100–200 ps. 

## 4. Conclusions

The studied regularities of interaction of SWCNTs with various electromagnetic waves in the range of 10–3000 nm showed that defective regions of SWCNTs absorb more energy than defect-free ones. The same effect is observed for SWCNT regions near their open ends. It was found that the absorption of the incident wave energy can reach 36%–40%. This allows us to make an assumption that nanowelding of SWCNTs and the formation of a branched SWCNT network will be carried out precisely in these regions, which will act as a hot-spot. Molecular dynamic modeling of SWCNT nanowelding showed that this process occurs rather quickly in time for several tens of picoseconds. The time of nanowelding with the formation of covalent bonds between SWCNTs is determined primarily by the distance between the contacting SWCNTs. The shorter this distance, the faster new bonds form.

The modeling of the formation of layered protein and polymer matrices based on SWCNTs, albumin, collagen, and chitosan showed that individual layers of these matrices are able to interact with each other. This interaction is energetically beneficial. This shows that such a layered biocompatible material is very promising in biomedical applications, in particular in tissue engineering. In silico studies of the interaction of protein and polymer matrices with membranes showed that the matrices do no harm to the cell membrane. The resulting small holes from the contacts with nanotubes are rapidly tightened within 100–200 ps. Some molecules of albumin, collagen, or chitosan that adhere to the membrane do not harm the membrane because they are completely biocompatible. In the future, mechanical properties of layered protein and polymer matrices with embedded carbon nanotubes will be considered.
